# Operational Insights From the Longitudinal Analysis of a Linear Accelerator Machine Log

**DOI:** 10.7759/cureus.16038

**Published:** 2021-06-29

**Authors:** Jeremy D Hoisak, Gwe-Ya G Kim, Todd F Atwood, Todd Pawlicki

**Affiliations:** 1 Radiation Medicine and Applied Sciences, University of California San Diego, La Jolla, USA

**Keywords:** halcyon, linear accelerator, machine log, quality management, fault recovery, incident learning systems

## Abstract

Purpose

This study aimed to perform a longitudinal analysis of linear accelerator (linac) technical faults reported with a cloud-based Machine Log system in use in a busy academic clinic and derive operational insights related to linac reliability, clinical utilization, and performance.

Methods

We queried the Machine Log system for the following parameters: linac type, number of reported technical faults, types of fault, number of faults where the linac was disabled, and estimated clinical downtime. The number of fractions treated and monitor units (MU) delivered were obtained from the record and verify system as metrics of linac utilization and to normalize the number of reported linac faults, facilitating inter-comparison. Two Varian TrueBeam C-arm linacs (Varian Medical Systems, Palo Alto, CA), one Varian 21iX C-arm linac (Varian Medical Systems, Palo Alto, CA), and one newly installed Varian Halcyon ring gantry linac (Varian Medical Systems, Palo Alto, CA) were evaluated. The linacs were studied over a 30-month period from September 2017 to March 2020.

Results

Over 30 months, comprising 677 clinical days, 1234 faults were reported from all linacs, including 153 “linac down” events requiring rescheduling or cancellation of treatments. The TrueBeam linacs reported nearly twice as many imaging, multileaf collimator (MLC), and beam generation faults per fraction, and MU as the Halcyon. Halcyon experienced fewer beam generation/steering, accessory, and cooling-related faults than the other linacs but reported more computer and networking issues. Although it employs a relatively new MLC design compared to the C-arm linacs and delivers primarily intensity-modulated treatments, Halcyon reported fewer MLC faults than the other linacs. The 21iX linac had the fewest software-related faults but was subject to the most cooling-related faults, which we attributed to extensive use of this linac for treatment techniques with extended beam-on times.

Conclusions

A longitudinal analysis of a cloud-based Machine Log system yielded operational insights into the utilization, performance, and technical reliability of the linacs in use at our institution. Several trends in linac sub-system reliability were identified and could be attributed to either age, design, clinical use, or operational demands. The results of this analysis will be used as a basis for designing linac quality assurance schedules that reflect actual linac usage and observed sub-system reliability. Such a practice may contribute to a clinic workflow subject to fewer disruptions from linac faults, ultimately improving efficiency and patient safety.

## Introduction

The medical linear accelerators (linacs) are complex systems that in addition to core systems related to beam generation and cooling to manage the associated thermal loads, including sub-systems like the multileaf collimator (MLC) [1] and volumetric imaging [2]. The modern linacs can also be dynamically linked to ancillary systems, such as robotic couches for remote patient positioning, and real-time motion monitoring with infrared, electromagnetic, and optical systems [3]. Integration of positioning and verification systems with the linac allows for precise and accurate treatment techniques such as intensity-modulated radiation therapy (IMRT), stereotactic radiosurgery (SRS), hypo-fractionated high-dose stereotactic body radiation therapy (SBRT), and respiratory-gated delivery. For intensity-modulated fields, the monitor units (MU) per treatment have also increased along with the complexity of techniques.

The healthcare equipment manufacturers are regulated and are incentivized to design the systems with multiple redundancies and interlocks to prevent linac performance deviations that can result in patient harm. However, linac faults routinely occur in normal operation without any harm to the patient. Some faults are designed to take the linac offline until the fault can be investigated and maintenance performed. Other faults are either quickly cleared or cleared after some action by clinic staff. This downtime can still have a clinical impact, particularly in a busy clinic, which can lead to treatment delays and time pressures on staff. Adapting to workflow disruptions in healthcare delivery can also increase the risk of error [4]. In radiation therapy, the errors originating in workflow disruptions may occur when moving the patients to a different linac or moving the staff who may not be as familiar with a patient’s setup and accessories [5].

With an aging population as well as the increased therapeutic use of radiation for malignant and benign diseases, the number of patients being treated is expected to increase [6]. Thus, the hardware and software performance demands on the linacs are increasing and their overall performance reliability will continue to be an important issue. Minimizing linac downtime and ensuring reliable patient throughput is therefore essential to providing high-quality radiation therapy. Furthermore, as radiation therapy becomes more widely available in low- and middle-income countries (LMICs), high throughput and reliability of radiotherapy equipment will be critical as service and support infrastructure may not be readily available [7].

Our institution employs a cloud-based electronic equipment fault reporting system (Machine Log) to alert the staff and vendor support of the incidence of faults, interlocks, and other technical issues originating with the linacs and other treatment-related equipment in the clinic. The Machine Log is compatible with any vendor or treatment equipment. The previous work has demonstrated that the Machine Log systems can provide operational efficiencies and reduce clinic disruptions, potentially improving patient safety and the quality of care [8]. The purpose of this work is to use a longitudinal analysis of Machine Log data for our institution’s linacs to perform a comparative evaluation of technical reliability and workflow disruptions, accounting for linac usage and patient throughput.

## Materials and methods

Our institution's principal clinic is equipped with four linacs of varying designs, ages, and clinical roles. These linacs are operated in a hospital setting and are supported by a vendor-provided technical help desk as well as field service engineers, although the latter are not based on site.

Linac descriptions

As shown in Table 1, these four linacs comprise two TrueBeam™ models (Varian Medical Systems, Palo Alto, CA) and one 21iX (Varian Medical Systems, Palo Alto, CA), all equipped with the Millenium™ 120-leaf MLC (Varian Medical Systems, Palo Alto, CA). The TrueBeam linacs are equipped with MV portal imaging, and kV-cone-beam computed tomography (CBCT). The 21iX was originally a 21EX model without imaging that was later upgraded with on-board imaging (OBI) several years prior to this study. Our institution is also equipped with a Varian Halcyon™ 2.0 (Varian Medical Systems, Palo Alto, CA). Halcyon is a novel ring-gantry linac configuration designed for streamlined operation and high patient throughput. Our institution was among the first to introduce the Halcyon into clinical service, installing the system in mid-2017 and treating the first patient in September 2017. This relatively new linac design consists of a single energy, magnetron-based 6 MV flattening filter-free (FFF) linear accelerator mounted on a rotating gantry with a beam stopper and electronic portal imaging device (EPID) mounted opposite. The maximum dose rate is 800 MU/minute. The EPID acquires either orthogonal MV image pairs or MV-cone-beam computed tomography (CBCT) images for setup verification. The MV-CBCT field of view is 28 cm x 28 cm with a 0.22 mm pixel spacing. At the time of commissioning and first clinical use, Halcyon did not have kV imaging; this capability was added approximately one year later in an upgrade to the Halcyon 2.0 standard. The kV-CBCT field of view is 28 cm by 28 cm with a 0.336 mm pixel spacing. Halcyon also has a novel MLC design that shapes the beam with a staggered, dual-layer leaf bank system and no collimator jaws. The upper layer consists of two banks of 29 leaves and the lower layer consists of two banks of 28 leaves, for a total of 114 leaves of 1.0 cm width. The maximum field size is 28.0 cm by 28.0 cm. The full gantry assembly is enclosed within a carbon fiber shell, surrounding a wide bore through which patients are positioned for the treatment. The linac rotates within the shell at a speed of four revolutions per minute (rpm), which is approximately four times faster than the rpm of conventional Varian linacs with a C-arm gantry design. The mechanical accuracy of the Halcyon MLC has been shown to be equivalent to the TrueBeam MLC while operating at a higher gantry rotation speed [9].

**Table 1:** Description of linacs, key features, and clinical use. FFF: flattening filter-free; CBCT: cone-beam computed tomography; SRS: stereotactic radiosurgery; SBRT: stereotactic body radiation therapy; VMAT: volumetric modulated arc therapy; TBI: total body irradiation; TSE: total skin electron *This linac was originally installed in another clinic in 2011 and re-located after three years. **kV imaging was added in an upgrade to Halcyon 2.0 in September 2018.

Linac Model	Installation Date	Approximate Age at Start of Study (years)	Clinical Energies		Imaging	Features and Clinical Use
			Photon (MV)	Electron (MeV)		
TrueBeam (TB1)	Oct. 2010	7	6, 6FFF, 15	6, 9, 12, 16, 20	kV, MV, kV-CBCT	SRS, SBRT, VMAT, gating
TrueBeam (TB2)	Nov. 2014	6*	6, 10FFF, 15	6, 9, 12, 16, 20	kV, MV, kV-CBCT	SRS, SBRT, VMAT, gating
21iX	May 2005	12	6, 15	6, 9, 12, 16, 20	kV, MV, kV-CBCT	3D, TBI, TSE
Halcyon	July 2017	0.25	6FFF	N/A	MV-CBCT, kV-CBCT**	VMAT

New technology can be associated with periods of higher service and support demands as installation and manufacturing issues become apparent, unknown design deficiencies appear, and staff build familiarity and develop competencies [10-12]. To yield operational insights into the initial technical reliability of Halcyon, we divided the 30-month evaluation period into two phases: the entry-into-service period of six months and subsequent 24 months of routine clinical use. Halcyon has been the subject of numerous evaluation studies, including characterization of the novel MLC design [13], acceptance and commissioning [14-16], output calibration [17], radiographic imaging [18-19], integration with surface imaging [20], and clinical safety [21]. 

Over the total evaluation period of 30 months, the clinic treated an average of 150 patients per day (range: 130-190). The typical treatment fraction durations were 15 minutes for conventional treatments and 30 minutes or longer for special procedures, such as stereotactic radiosurgery (SRS) and stereotactic body radiation therapy (SBRT), delivered exclusively on the TrueBeam linacs or total body irradiation (TBI) and total skin electron (TSE) therapy delivered exclusively on the 21iX linac.

Linac Machine Log system

Although the most modern linacs have some ability to automatically record faults and other errors internally, this data is not readily available to clinic staff, often lacks context about how the linac was being used at the time and the clinical impact of the fault. Our institution employs a cloud-based Machine Log system to record all the technical faults arising from the linacs, regardless of significance or downtime, in the form of a user-generated context-rich report. The Machine Log also serves as an institution-wide event notification and management system for disseminating and storing linac fault reports. The clinic staff including radiation therapists and medical physicists can report faults via a simple webpage with a box for free-text entry, predefined checkboxes for event categorizing, and radio buttons to indicate linac status. Machine Log reports also quantify linac downtime and provide the option to electronically notify the vendor and request technical help desk or field service engineer support. The system is designed for ease and speed of entry so that reports can be distributed in near real-time. The user review of Machine Log report entries is performed from any web browser including mobile devices and can be filtered by linac, date/time, linac status (i.e., down), event resolution status, and is keyword searchable. The events can be updated or marked as resolved with follow-up information, such as repair information. An in-house browser-based version of the Machine Log system was used for the first 18 months of data collection before we transitioned to the functionally similar cloud-based total QA Machine Log system (Image Owl, Inc., Greenwich, NY) for the remaining 12 months of the study.

Linac Machine Log longitudinal analysis

Between September 2017 and March 2020, the Machine Log was queried for the following parameters: linac type, number of reported faults, fault descriptions, and clinical downtime. From the reported faults and associated descriptions, events that resulted in a “linac down event” (LDE) were identified and separately tabulated. An LDE was defined as a fault with the linac that interrupted or prevented the patient's treatments and required cancellations or adjustments to the linac schedule. The faults that result in an LDE can be classified as such in the Machine Log through a dedicated checkbox, or if the keywords “MACHINE DOWN” or “LINAC DOWN” were present in the free text of the report. The reported downtime per fault was estimated by clinical staff from the time of the initial report to return to service. The record and verify (R&V) system (Aria Oncology Information System, Varian Medical Systems, Palo Alto, CA) was used to determine how many patients were rescheduled or canceled after an LDE or other significant fault. The R&V was also queried to cross-validate the staff estimates of clinical downtime by inspecting the appointment schedule at the date and time of an LDE. The fault reports were classified into one of eight categories representing major sub-systems present on each linac: imaging systems (including kV, MV, and CBCT), MLC, beam generation (including vacuum), cooling systems, software/network, patient support, accessories (including electron applicators, light field/optical distance indicator and related setup aids), and miscellaneous faults falling outside these categories.

The start of the analysis (September 2017) coincided with the post-commissioning deployment of the new Halcyon linac into routine clinical use. Therefore, in addition to the full 30-month analysis, we performed a separate analysis on a six-month subset of data (September 2017-March 2018), where we expected higher technical support demands for Halcyon as quality issues were resolved and staff became familiar with the system’s abilities and demands. During this period, clinical use was also constrained by anatomic sites as users and clinicians developed more confidence in the system’s capabilities. The results of this subset analysis were compared with the overall 30-month analysis, where clinical use of Halcyon expanded both in the patient numbers and sites treated, and several hardware and software upgrades were made. The goal of this comparison was to identify the technical reliability and patient throughput trends and other operational insights related to the deployment of a new treatment unit.

Linac clinical utilization

The R&V system was queried for the number of fractions treated and MU delivered as metrics of linac utilization. To enable a meaningful cross-comparison between linacs with varying patient loads, clinical use profiles, and capabilities, the number of reported faults per sub-system was normalized to the number of fractions delivered and to the number of monitor units (MU) delivered. For ease of data presentation, the metric was expressed as the number of reported faults per 1000 fractions delivered and the number of faults per 1x10^6^ MU delivered. We considered the number of fractions delivered by a linac to be a reasonable surrogate for the level of demand placed on sub-systems that are used intermittently in a treatment fraction, i.e., before and after the treatment, such as the patient support, imaging, and control interfaces. Conversely, we considered the number of MU delivered by a linac to be a surrogate for the level of demand placed on the core systems of the linac that are in more continuous operation during a treatment fraction, such as cooling, beam generation, steering, and collimation. Other metrics of linac clinical utilization and patient load were calculated relative to the number of fractions treated and MU delivered, respectively, in order to account for the different clinical uses of the linacs.

## Results

Halcyon performance was evaluated at six months from the start of clinical service and at 30 months from the start of clinical service. During the initial six-month period, the anatomic sites treated with Halcyon were limited as staff developed familiarity with the capabilities and performance of the linac.

Initial six-month evaluation of Halcyon clinical use

The initial six months of Halcyon clinical service comprised 119 clinical days. In this time, Halcyon delivered 20.1% of all fractions treated in the clinic, reflecting the reduced usage compared to the other linacs in the clinic (Table 2). In this same period, Halcyon accounted for 30.3% of the MU delivered in the clinic, and delivered the most MU per fraction, reflecting the heavier reliance on intensity modulation with this linac type. Halcyon reported 15.6% of all recorded linac faults, 21.7% of all LDEs, and 18.4% of all clinical downtime. The number of MU delivered between faults and between LDEs was also the highest for Halcyon. The number of fractions delivered per fault on Halcyon was comparable to the 21iX.

**Table 2:** Comparison of linac workload measured in numbers of fractions and MU, numbers of faults and LDE, and clinical downtime during Halcyon’s first six months of clinical service. (Values in brackets indicate the fraction of the clinic total, in percent.) MU: monitor units; LDE: linac down events

	TrueBeam 1	TrueBeam 2	21iX	Halcyon
Number of fractions delivered	4382 (23.8)	5270 (28.7)	5044 (27.4)	3698 (20.1)
Number of fractions/day	36.8	44.3	42.4	31.1
Number of MU delivered	6.7×10^6^	5.8×10^6^	3.9×10^6^	7.2×10^6^
Number of MU/fraction	1538	1100	777	1935
Number of faults	126 (31.2)	131 (32.4)	84 (20.8)	63 (15.6)
Number of LDE	7 (30.4)	6 (26.1)	5 (21.7)	5 (21.7)
Clinical downtime (min)	725 (28.9)	857 (34.2)	463 (18.5)	461 (18.4)
Number of fractions/fault	34.8	40.2	60.0	58.7
Number of MU/fault	0.54×10^5^	0.44×10^5^	0.47×10^5^	1.1×10^5^
Number of MU/LDE	9.6×10^5^	9.7×10^5^	7.8×10^5^	14.3×10^5^

**Figure 1:**
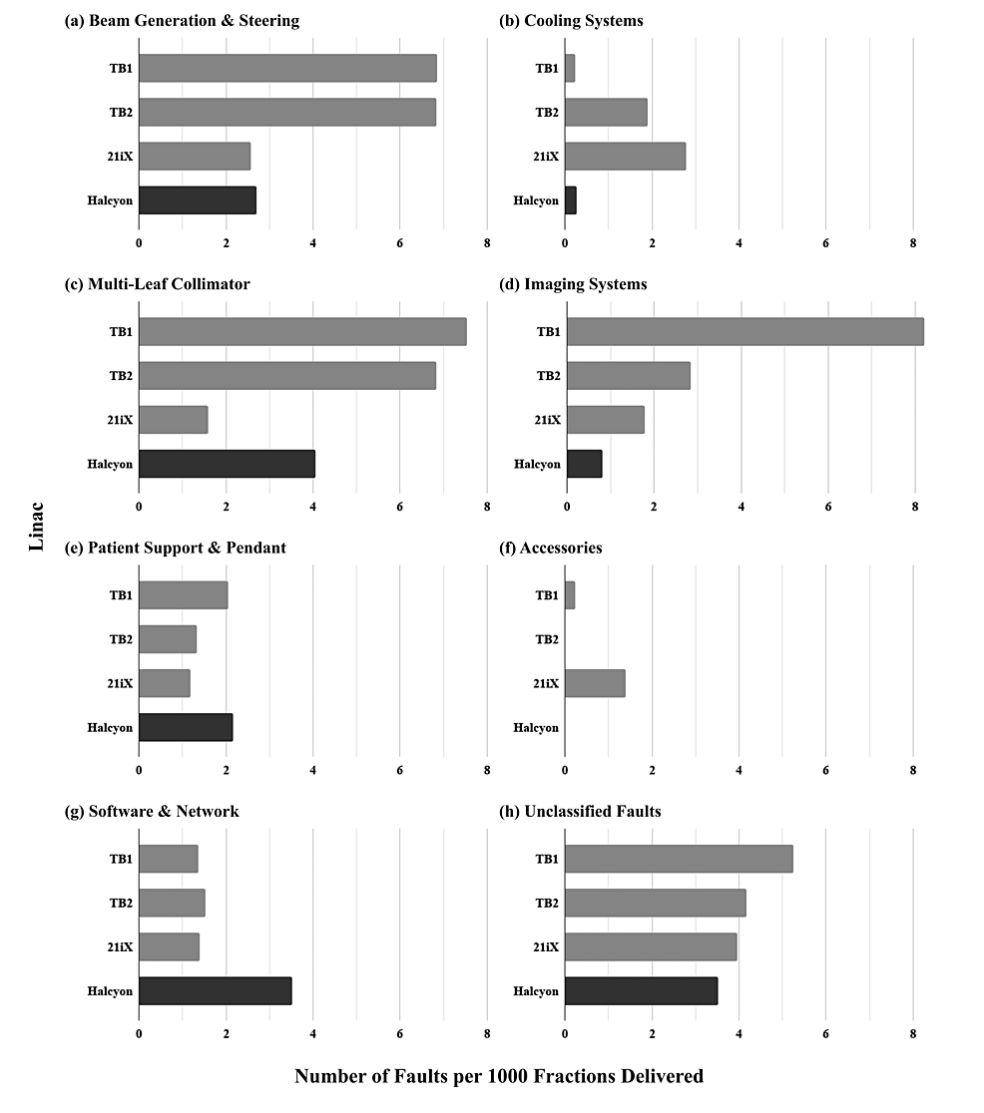
Comparison of the rate of linac faults per 1000 fractions delivered, organized by linac and sub-system fault over the initial six months of Halcyon clinical service. (Halcyon results are highlighted with a darker shade.)

Normalized per 1000 fractions delivered by each linac (Figures 1a-1h), the TrueBeam linacs reported more faults related to beam generation, imaging, and the MLC (Figures 1a, 1c, 1d). Halcyon reported fewer MLC-related faults than the TrueBeam linacs, but more than the 21iX (Figure 1c). The 21iX reported the most faults related to cooling systems (Figure 1b) and the fewest faults related to the patient support/pendant (Figure 1e). Accessories faults were almost exclusively reported by the 21iX (Figure 1f). Halcyon reported the most software/network-related faults (Figure 1g) and patient support-related faults (Figure 1e). 

Thirty-month evaluation period

After 30 months of clinical service (inclusive of the initial six-month period), or 677 clinical days, Halcyon had assumed a larger role in the clinic, delivering 30.8% of all fractions, and demonstrated the highest patient throughput, with an average of 43 fractions delivered per day (Table 3). Over this period, Halcyon accounted for 17.4% of all reported linac faults, 13.7% of all LDEs, and 11.2% of all clinical downtime among the clinic’s four linear accelerators.

**Table 3:** Comparison of linac usage measured in numbers of fractions and MU, numbers of faults and linac down events LDE, and clinical downtime over 30 months of clinical service. (Values in brackets indicate the fraction of the clinic total, in percent.) MU: monitor units; LDE: linac down events

	TrueBeam 1	TrueBeam 2	21iX	Halcyon
Number of fractions delivered	19,115 (20.2)	22,511 (23.8)	23,743 (25.1)	29,108 (30.8)
Number of fractions/day	28.2	33.3	35.1	43.0
Number of MU delivered	4.31×10^7^	3.44×10^7^	2.00×10^7^	5.68×10^7^
Number of MU/fraction	2252.4	1527.9	840.7	1951.2
Number of faults	370 (30.0)	394 (31.9)	255 (20.7)	215 (17.4)
Number of LDE	47 (30.7)	52 (34.0)	33 (21.6)	21 (13.7)
Clinical downtime (min)	7164 (48.7)	4299 (29.2)	1593 (10.8)	1647 (11.2)
Number of fractions/fault	51.7	57.1	93.1	135.4
Number of MU/fault	1.2×10^5^	0.87×10^5^	0.78×10^5^	2.6×10^5^
Number of MU/LDE	9.22×10^5^	6.6×10^5^	6.1×10^5^	27.0×10^5^

**Figure 2:**
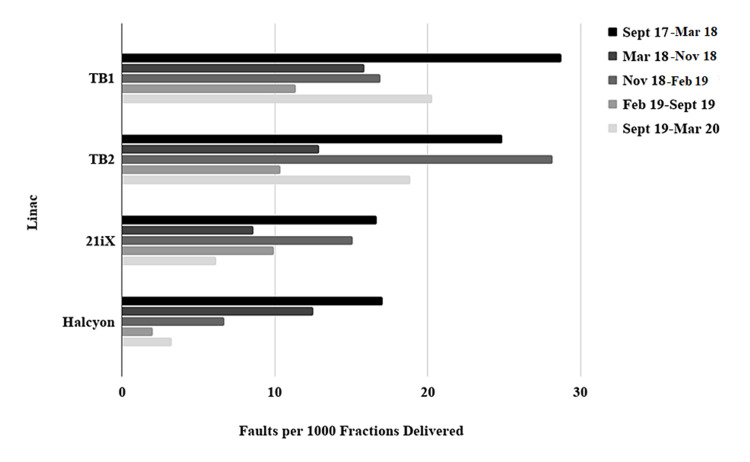
Comparison of an overall linac technical reliability over 30 months of clinical service expressed as the number of reported faults per 1000 fractions delivered by each linac. Time intervals demonstrate linac fault reliability over various periods of clinic throughput and patient load on each linac.

When the number of faults and LDE were normalized to the number of fractions and MU delivered, the Halcyon experienced less than half of the rate of faults per MU and, on average, less than one-third of the rate of LDE per MU when compared with the other linacs. There was a decrease in reported faults per 1000 fractions delivered after the six-month introductory period for Halcyon had ended in March 2018 (Figure 2). The other linacs also exhibited a decrease in the faults per 1000 fractions delivered in the same period. This coincided with a 38% increase in fractions delivered per day on the Halcyon, and reductions ranging from 27% to 33% in patients treated per day on the other linacs (Tables 2, 3).

**Figure 3:**
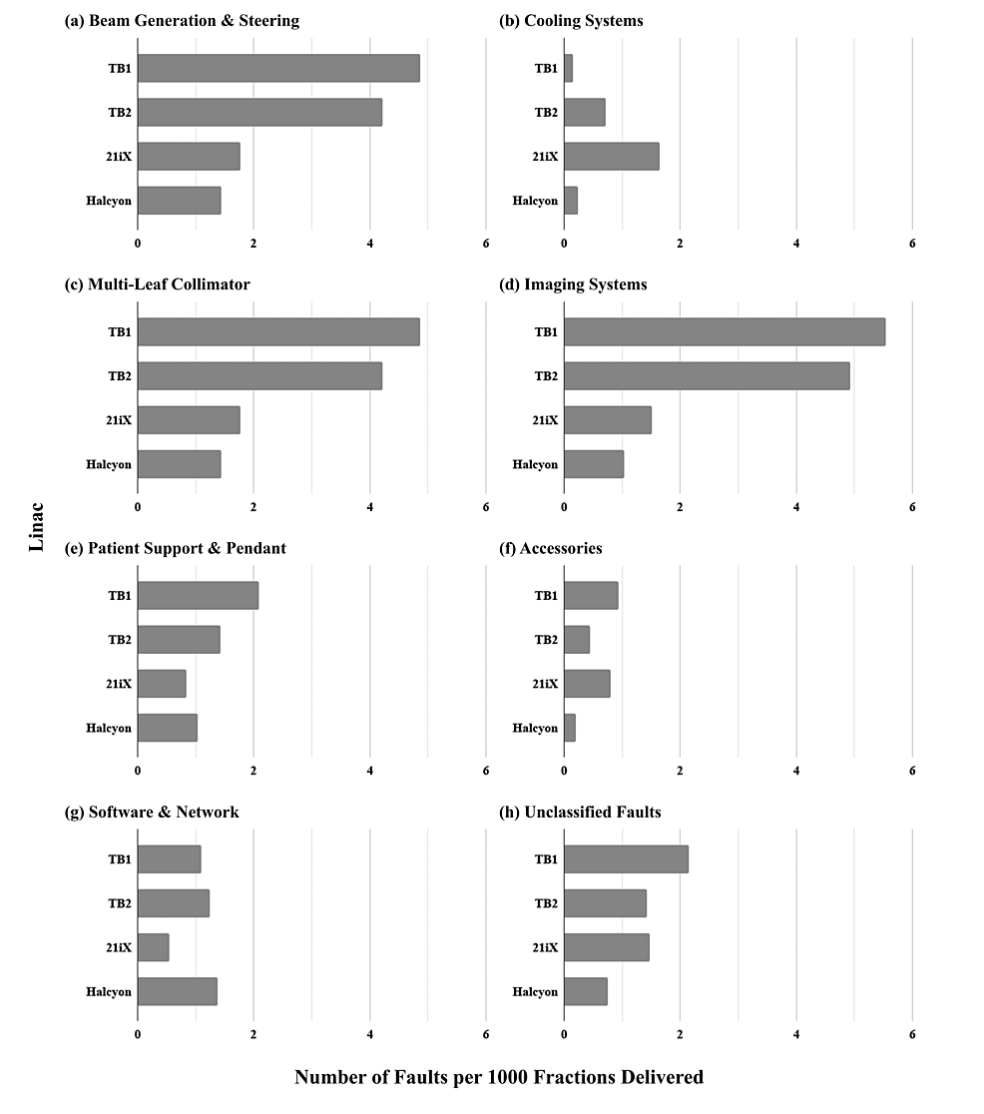
Comparison of the rate of linac faults per 1000 fractions delivered, organized by linac and sub-system fault over 30 months of clinical service (inclusive of the initial six-month Halcyon evaluation period).

Over the subsequent 24 months (30 months since introduction), the absolute numbers of faults on Halcyon increased coincident with the period of expanded clinical utilization and an associated increase in patient throughput (Table 3). However, when normalized to the number of fractions delivered (Figures 3a-3h), the trends in Halcyon reliability continued, with a lower frequency of faults compared to the other linacs in beam generation, MLC, imaging, and accessories (Figures 3a, 3c, 3d, 3f). Faults related to the patient support (Figure 3e) and software/network seen in the initial six-month period continued to be relatively higher than with the other linacs (Figure 3g). 

**Figure 4:**
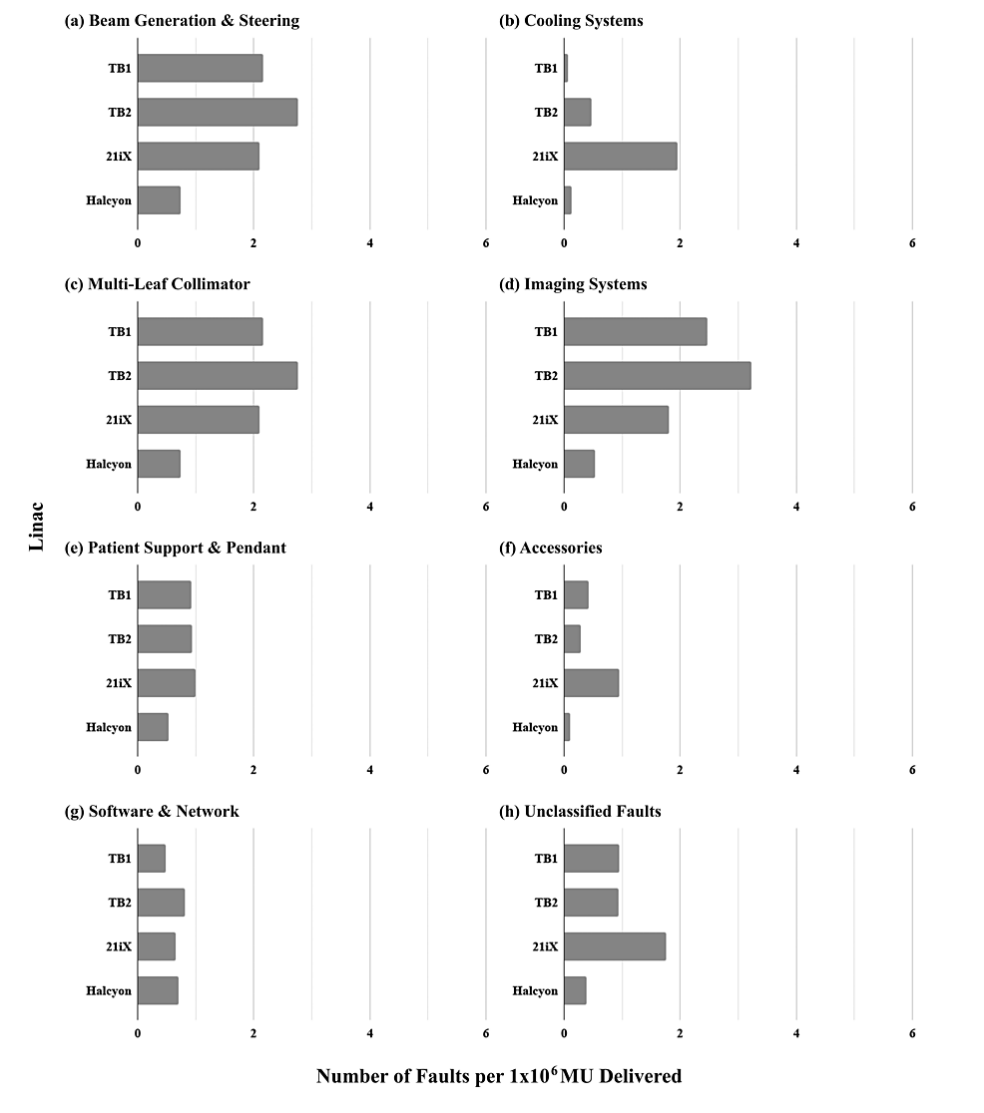
Comparison of the rate of linac faults per 1x10^6 MU delivered, organized by linac and sub-system fault over 30 months of clinical service (inclusive of the initial six-month Halcyon introductory period). MU: monitor units

When normalized by the number of MU delivered by each linac (Figures 4a-4h), Halcyon demonstrated the lowest frequency of faults in systems related to output including beam generation (Figure 4a) and MLC (Figure 4c), and was roughly equivalent to the TrueBeam 1 for the lowest frequency of faults in cooling systems (Figure 4b). When normalized by MU, the high frequency of cooling-related faults on the 21iX is further emphasized (Figure 4b). Fault frequency for sub-systems not directly related to output became more similar across linacs when normalized by MU instead of by fraction, including the patient support and software/network (Figures 4e, 4g).

## Discussion

In radiation oncology, much emphasis has been placed on creating the treatment delivery systems and clinical processes that maximize equipment, human, and organizational reliability. The American Association of Physicists in Medicine (AAPM) report of Task Group 100 recommends risk assessment methods, such as Failure Modes and Effects Analysis and Fault Tree Analysis, to identify and develop the strategies to mitigate the risk of errors arising from human and systems failures [22]. The real-time equipment fault reporting and electronic incident learning systems are another way of mitigating risk by providing a feedback mechanism for quality improvements to both clinical users and vendors. Electronic fault reporting databases such as Machine Log can be analyzed for linac performance and technical reliability and yield insights into machine performance and clinical throughput. The longitudinal analysis of linac faults presented here revealed several notable observations and trends regarding linac technical reliability and clinical availability.

Halcyon initial clinical deployment and operational insights

While some new equipment installations can be problematic, despite having a new linac design including novel MLC design and user interface, Halcyon was at least as reliable as conventional Varian linac designs during the initial six-month clinical deployment. Over a subsequent 24 months of clinical service (30 months total), Halcyon delivered more fractions and more MU per reported faults and LDE than either the TrueBeam linac or the 21iX design. Halcyon faults related to beam generation, beam steering, and cooling systems occurred less frequently than with the other linacs. This may be because the Halcyon employs a single photon energy design compared to the multiple energies and electron/photon modes available on the C-arm linacs. At the same time, Halcyon delivered the most MU, in absolute terms and relative to the number of fractions and reported faults, suggesting a core systems design that is robust to high treatment workloads.

Halcyon also reported the fewest MLC faults per MU and per fractions delivered. This is also noteworthy as Halcyon employs more intensity modulation than the C-arm linacs, potentially placing more mechanical stress on the system. The technical reliability of the Halcyon MLC is a finding generally consistent with Wang et al., who reported that the mechanical accuracy and reproducibility of the Halcyon MLC was superior to that of the TrueBeam MLC [9].

A major design goal of Halcyon was high patient throughput with reduced service and support requirements [23]. Our findings indicate that Halcyon had less clinical downtime and fewer LDE compared to linacs with comparable patient throughput, demonstrating that the Halcyon facilitates high patient throughput with reduced service and support requirements. From the low clinical downtime relative to the number of faults, it can also be deduced that most Halcyon faults were quickly recoverable either by clinic staff acting independent of vendor support or with the assistance of remote technical support, and did not require long waits for vendor field service engineers to arrive on the site to resolve the event. While laudable in their own right, meeting these design goals would also make Halcyon suitable for deployment in resource-limited environments, including LMICs. A comparative analysis of linac reliability in the United Kingdom and Africa found that linac failures were significantly higher in LMICs, with the MLC and cooling systems responsible for the highest rates of faults [7]. In this work, Halcyon reported the lowest rate of MLC faults and among the lowest rate of cooling system faults. A further benefit of having a highly reliable treatment unit in the clinic is shown in Figure 2, where all linacs exhibited a decrease in faults per fraction delivered after the initial six-month deployment of Halcyon had ended. We attribute this phenomenon to the reduction in patients treated per day on all non-Halcyon linacs as our institution gained confidence and experience with Halcyon and increased the patient load on the new linac. This redistribution of patient load reduced the demands placed on all other linacs and their subsystems, which may have contributed to a lower frequency of technical faults.

Imaging systems on Halcyon proved to be reliable compared with the other linacs at our institution. In the initial six months of service, the Halcyon reported fewer imaging-related faults than the conventional linacs, however, this can be partly explained by the relatively simple imaging systems in place over this period compared to the other linacs in our department. Halcyon had MV-CBCT only at the time of commissioning and during the first year of operation. Kilovoltage X-ray imaging capabilities including CBCT were not added to Halcyon until after one year of service when the system was upgraded to the Halcyon 2.0 standard. However, even when adding additional imaging systems, Halcyon still reported fewer imaging-related faults per 1000 fractions delivered (Figure 3d) and per 1x10^6^ MU when compared with the other linacs (Figure 4d). We believe that this may be partly due to the less complex mechanical design of the kV imaging system on Halcyon, in which the source and detector are fixed to the gantry and do not require multiple moving arms to position them for imaging as with the C-arm linac OBI system, a common source of faults in our experience with the other OBI-equipped linacs in our clinic.

The software and networking-related faults were one area where Halcyon reported more faults than the other linacs in our institution, both during the initial six-month introduction to the clinical service and over the full evaluation period (Figures 3g, 4g). As both the maturity of the software and the experience of staff in managing the network configuration grew over time, faults of this nature were expected to decrease. Indeed, some software-related issues were addressed in the upgrade to Halcyon 2.0 when kV imaging hardware was also added. However, software-related issues persisted. Such faults may depend on information technology infrastructure and may be institution-specific; a comparison with other user experiences is needed to determine if software and networking faults are a common problem with Halcyon. The Halcyon also exhibited a recurring issue with the patient support that became notable in the second year of service but was resolved through a vendor-provided hardware fix.

TrueBeam operational insights

The imaging systems (MV portal imager and kV OBI system) were the greatest source of faults for both TrueBeam linacs in our clinic. Imaging system faults were also more frequently reported compared to the 21iX and Halcyon linacs (Figures 3d, 4d). At our institution, the TrueBeam linacs are primarily used for imaging-intensive procedures, such as SRS and SBRT. It remains unclear if the higher frequency of imaging system faults on TrueBeam linacs is a factor of their design or their more intensive clinical utilization compared to the other linacs.

Beam generation was the next most common source of faults (Figures 3a, 4a). TrueBeam linacs have multiple energies including FFF modes, and these linacs are employed to deliver respiratory-gated treatments. The associated demands of these capabilities on the beam generation system could explain the higher beam generation fault rates compared with Halcyon, which is a single energy (6 MV) machine. However, the TrueBeam linacs reported 34 faults related to electrons or photon energies other than 6 MV over the 30-month evaluation period, out of a total of 764 TrueBeam linac faults (4.4%). This low number is not unexpected as most fractions in the IMRT era are delivered with 6 MV photons. However, the low proportion of non-6 MV-associated faults suggests that having multiple energies was not a significant factor in this analysis of linac technical reliability.

The MLC on the TrueBeam linacs was the third greatest source of faults per fraction delivered (Figure 3c). Again, this likely reflects their primary clinic role in delivering high-dose intensity-modulated treatments, such as SRS and SBRT. Indeed, when normalized by MU delivered, the frequency of MLC faults becomes more comparable to the 21iX linac, which has the same MLC design (Figure 4c).

21iX operational insights

The 21iX is the oldest linac in our clinic and is the only linac used to delivery TBI and TSE treatments. These two techniques require extended beam-on times and generate correspondingly higher thermal loads in the linac. Therefore, reliable cooling systems are essential to treating patients without interruption. It follows that reliable cooling systems are essential to treating patients without interruption. This analysis showed that the greatest source of faults for the 21iX originated with the cooling systems when corrected for usage (Figures 3b, 4b). The 21iX reported the fewest software and network-related faults, which are expected due to the older and less complex control software in use at the treatment console relative to the TrueBeam linacs, as well as the maturity of the network configuration relative to the more recent Halcyon installation (Figures 3g, 4g). When not used for TBI or TSE, our institution’s 21iX primarily delivers three-dimensional conformal radiation therapy (3D-CRT), corresponding to lower demand on the MLC and imaging systems compared to the TrueBeam and Halcyon linacs. Consequently, the 21iX exhibited a low rate of imaging and MLC-related faults. Relative to the other linacs, the patient support system on the 21iX reported the fewest faults per 1000 fractions delivered (Figure 3e). As with imaging and use of the MLC, we believe this is because the treatments delivered on the 21iX rely less on automatic couch movements for image-guided radiation therapy (IGRT), or do not use the couch at all to support the patient, as our institution’s current technique for delivering TBI uses an extended source to surface distance (SSD) with the patient on a gurney. When normalized to MU delivered, the 21iX patient support exhibited similar technical reliability as the other C-arm-based linacs (Figure 4e).

Towards usage-based quality assurance intervals

In this study, linac performance and reliability were evaluated by comparing linac fault frequency as a function of delivered MU and of fractions treated to account for the different clinical use profiles of each linac. Expressing fault frequency in terms of MU and fractions delivered also yielded possible explanations for the observed technical reliability of some sub-systems. For example, the 21iX was the oldest linac in our clinic, but reported the fewest faults in absolute numbers of the conventional linac designs and delivered the most fractions without a fault. However, when evaluated per MU delivered, the 21iX delivered the fewest number of MU per fault. This suggests that for this linac, although some ancillary subsystems had high technical reliability, core linac systems such as beam generation, collimation and cooling may require increased maintenance attention. It has been proposed that age could define the optimum maintenance strategy for medical equipment, with more technologically advanced systems receiving predictive maintenance strategies while older systems receive more traditional preventive maintenance [24]. 

The vendor service engineers are a limited resource in the clinic, often supporting multiple machines at multiple institutions within a geographic region. To address this challenge efficiently, many industries have shifted their preventive maintenance (PM) strategies from calendar-based to those based on a continuous device lifespan-based schedule, which is both more effective and more efficient [25]. The development of similarly efficient quality assurance (QA) strategies to maintain linac quality and safety will allow clinical medical physicists to contribute more directly to patient care including online adaptive therapy [26], patient communication and education [27], and other new roles as described by the Medical Physics 3.0 initiative [28]. The results of this study suggest another QA strategy where intervals are shifted away from calendar-based (i.e., weekly, monthly, annual) schedules towards ones informed by the generalized linac fault trends and known sub-system problem areas for each linac and vendor, such as can be identified through a Machine Log analysis. For example, the analysis of our institutions’ linacs indicates that a customized PM and QA strategy for our clinic could emphasize imaging systems and the MLC on the TrueBeam linacs and place less emphasis on MLC QA for the Halcyon. The relationship between linac faults and MU and fractions delivered indicates that the intervals for this new QA approach should be based on usage not the time since the last test. Our analysis indicated that faults per MU and per fraction decreased when relative patient loads on the linacs were reduced, suggesting that a usage-based PM and QA schedule is more appropriate than one that is purely calendar-based. A robust QA strategy would be to combine a usage-based approach with a daily approach that is targeted at monitoring subsystems that are more likely to fail over time. The potential benefits to clinic efficiency could be investigated by repeating the analysis presented in this work, and quantifying linac performance before and after implementation of the new QA strategy.

Limitations

A limitation of this work is that the Machine Log system presented here is based on voluntary staff participation to compose and distribute reports. As a result, there is the possibility that some faults were simply cleared and not reported, particularly in time-constrained situations. However, our institution has a high participation and reporting rate thanks to the streamlined and user-friendly web browser interface for reporting and following up on linac events. Automatic fault reporting by the linac would be a solution to this problem, and this data could potentially be used to predict downtime [29] or the need for QA [30]. However, automatic reporting by the linac itself on faults would not provide the rich contextual information that is often beneficial in troubleshooting present and future linac problems. Another limitation of this work is that it compares different linac designs with further differences in the ages, capabilities, and clinical uses of each linac. We addressed these differences by comparing faults from similar sub-systems, and by normalizing fault frequencies with the numbers of fractions delivered and MU delivered. From Table 3 and Figures 3, 4, there was no obvious correlation between linac age and frequency of faults by MU or by a fraction. Furthermore, we observed only a small proportion of faults (6.3%) attributable to features not found on all linacs, thus we did not consider capability differences alone to be a factor in the superior or inferior reliability performance of some linacs.

A more general limitation of this analysis is that the findings reflect a single institution’s experience with these linacs and the fault trends for their respective sub-systems. A comparison against the experience of other institutions deploying these linac designs will be required to validate these findings and help determine which fault trends are installation-specific or possibly attributable to the linac design. The Machine Log analysis demonstrated here is a generalizable method for analyzing linac performance and can be applied to other institutions and delivery systems to investigate their comparative performance.

## Conclusions

A longitudinal analysis of data from a linac fault reporting system identified several trends and operational insights related to linac reliability, performance, and clinical usage. Over a 30-month routine use in a busy academic institution, the new Halcyon linac design proved at least as reliable as the well-characterized conventional linacs in our clinic and may be more reliable in some critical sub-systems related to beam generation, multi-leaf collimation, and imaging. The longer-term evaluation and comparison with other Halcyon installations would be necessary to determine if these findings and trends persist. The C-arm linacs exhibited varying technical reliability trends by sub-systems including imaging, MLC, and cooling. Other trends became apparent when normalized by MU delivered and fractions treated. The future study will employ this analysis to investigate if shifting QA and preventive maintenance schedules away from calendar-based intervals and towards intervals that reflect actual linac usage and observed technical reliability contributes to a clinic workflow that is subject to fewer equipment-related disruptions, improving efficiency and patient safety.
